# Comparative analysis of automated foul detection in football using deep learning architectures

**DOI:** 10.1038/s41598-025-96945-0

**Published:** 2025-04-24

**Authors:** Abdallah Rabee, Zakaria Anwar, Ahmed AbdelMoety, Ahmed Abdelsallam, Mahmoud Ali

**Affiliations:** 1 Sports Health Sciences Department, Faculty of Physical Education, South Valley University, Qena, 1464091 Egypt; 2 Sports Training and Kinesiology Sciences Department, Faculty of Physical Education, Suez University, Suez, 43512 Egypt; 3 Electrical Engineering Department, Faculty of Engineering, South Valley University, Qena, 83523 Egypt

**Keywords:** Deep learning evaluation, Foul detection, Image recognition, Sports analytics, Engineering, Computer science

## Abstract

Automated foul detection in football represents a challenging task due to the dynamic nature of the game, the variability in player movements, and the ambiguity in differentiating fouls from regular physical contact. This study presents a comprehensive comparative evaluation of eight state-of-the-art Deep Learning (DL) architectures — EfficientNetV2, ResNet50, VGG16, Xception, InceptionV3, MobileNetV2, InceptionResNetV2, and DenseNet121 — applied to the task of automated foul detection in football. The models were trained and evaluated using a curated dataset comprising 7000 images, which was split into 70% for training (4,900 images), 20% for validation (1,400 images), and 10% for testing (700 images). To ensure fair evaluation, the test set was balanced to contain 350 images depicting foul events and 350 images representing non-foul scenarios, although perfect balance was subject to class distribution constraints. Performance was assessed across multiple metrics, including test accuracy, precision, recall, F1-score, and Area Under the Receiver Operating Characteristic Curve (AUC). The results demonstrate that InceptionResNetV2 achieved the highest test accuracy of 87.57% and a strong F1-score of 0.8966, closely followed by DenseNet121, which attained the highest precision of 0.9786 and an AUC of 0.9641, indicating superior discriminatory power. Lightweight models such as MobileNetV2 also performed competitively, highlighting their potential for real-time deployment. The findings highlight the strengths and trade-offs between model complexity, accuracy, and generalizability, underscoring the viability of integrating DL architectures into existing football officiating systems, such as the Video Assistant Referee (VAR). Furthermore, the study emphasizes the importance of model explainability through techniques such as Gradient-weighted Class Activation Mapping++ (GradCAM++), ensuring that automated decisions can be accompanied by interpretable visual evidence. This comparative evaluation serves as a foundation for future research aimed at enhancing real-time foul detection through multimodal data fusion, temporal modeling, and improved domain adaptation techniques.

## Introduction

Among the most watched sports globally, football draws a lot of spectators and significantly affects both fan engagement and the local economy^1–3^. The dynamic architecture of the game, which is characterized by continuous action and complex player interactions, makes it difficult for officials to exactly spot and fix violations in real-time^4^. While the Video Assistant Referee (VAR) has been brought in to help with decision-making in football matches, human errors and oversight still exist and lead to divisive opinions that might influence game outcomes^5^. Consequently, more consistent and automated solutions to help officials in making accurate and quick decisions are becoming more and more needed^6^.

### Advances in technology

Advances in Computer Vision (CV) and Deep Learning (DL) have opened chances for the construction of sophisticated automated systems to identify fouls^7^. These systems can quickly and consistently assess large amounts of visual data, identifying intricate trends and anomalies that could be undetectable to human view^8^. Modern DL architectures allow one to design systems that can not only detect fouls but also provide instantaneous feedback, hence enhancing the general fairness and efficiency of the game^9^.

Recent studies stress player detection as a forerunner of foul detection. Deep Convolutional Neural Networks (CNNs) were one method applied to identify players across many spatial resolutions, hence improving identification accuracy in films of different quality^4^. Finding questionable contact that results in fouls depends on consistent player tracking.

Combining multi-object tracking and spatiotemporal action detection, a noteworthy work offered a DL pipeline to detect players engaged in fouls. Even in congested game footage, the algorithm precisely noted which players fouled and which committed fouls^10^.

DL techniques have made significant progress in image processing in various domains from business to healthcare. Based on finely described patterns in medical pictures, recent works have indicated that CNNs and Vision Transformers (ViTs) are superior to conventional techniques when distinguishing complex illnesses such as cerebral vascular occlusions and skin cancer^11–13^. For early skin cancer diagnosis, hybrid CNN-ViT models have, for instance, enhanced generalizability and accuracy even on unbalanced datasets^11^. ViT-based models have also shown almost complete accuracy in cervical cancer screening, above traditional CNNs^14^. These developments show how well DL can handle jobs with fine-grained visual knowledge, including football’s automatic foul identification. Techniques include attention mechanisms, domain-specific augmentation, and explainability tools like Gradient-weighted Class Activation Mapping++ (GradCAM++) help foul detection models to become both accurate and interpretable, hence improving their utility in real-time officiating systems such as VAR technology.

### Research focus

This research makes it possible to do a quantitative research about the effectiveness and comparative performance of various state-of-the-art DL architectures for the task of automatically detecting fouls in football matches. Standard football foul detection methods depend significantly on human perception, comprising real-time referee selections during the match or through post-match inspection via video technologies such as VAR systems. Although these approaches leverage the expertise of human interpretation—they are subject to inconsistency, bias and error, especially in dynamic or uncertain situations.

This study investigates the use of DL-based image analysis as a means of increasing the objectivity, consistency and efficiency of foul detection. It uses an annotated collection of football match images with bounding boxes representing foul incidents as a ground truth dataset to generate a binary classifier competent of discriminating whether a particular point in a match indicates a foul or not. It also seeks to explore how various architectures of Convolutional Neural Network (CNN) such as EfficientNetV2, ResNet50, VGG16, Xception, InceptionV3, MobileNetV2, InceptionResNetV2, and DenseNet121 process the anomalies of the real-world foul detection problem including variation in player positions, angle of camera perspective, light conditions, partial occlusion, among others.

Along with classification performance, this study also explores the explainability of these models through GradCAM++, which is an interpretability approach that allows us to visualize the regions of an image that were critical to its final output. Such dual emphasis on interpretability and performance relates to the wider target of embedding automated foul detection into commercial VAR systems, where accurate and understandable decision support are essential. By conducting a detailed comparative analysis, this research seeks to offer a well-rounded exposition of various DL strategies for ideal deployments in football officiating technologies, and their respective strengths and limitations.

### Research objectives

The focus of this research lies in designing and analyzing a sophisticated automated approach for detecting if an event was a foul or not through the use of DL methods on football matches. The objective of this study is to explore the comparison of performance of eight existing popular DL architectures like EfficientNetV2, ResNet50, VGG16, Xception, InceptionV3, MobileNetV2, InceptionResNetV2, and DenseNet121 to resolve the complex challenges posed by foul detection in dynamic conditions of a football match. This adds a level of complexity to accurately identifying foul events, considering the diverse scenarios in which such events occur, such as horizontal and vertical player positioning, different camera angles, diverse lighting conditions and occlusions. Using an annotated dataset containing images from football matches, where fouls were annotated, the research aims to study the generalization capacity of each model on training, validation, and balanced test sets. This study also includes explainability techniques such as GradCAM + + to produce visual heatmaps to find the significant regions of the image affected each model decision apart from the evaluation of classification performance through important metrics such as accuracy, precision, recall, F1-score, and Area Under the Receiver Operating Characteristic Curve (AUC). This attention to both predictive accuracy and interpretability is critical for achieving these goals, and is a necessary step towards ensuring that any the functioning system will not only have the potential to successfully identify fouls, but also, if desired, provide referees and video analysts with transparent visual justifications behind the system’s outputs. Additionally, the study will assess each model’s computational efficiency, stability, and practical feasibility for real-time use in current VAR systems. Finally, this study hopes to make a contribution towards developing a fair and consistent foul detection system, providing improved accuracy and interpretability in officiating decisions during professional soccer games.

This research paper is organized into several sections to comprehensively present the development, implementation, and evaluation of deep learning architectures for automated foul detection in football. Following the "Introduction", which outlines the background, technological advances, research focus, and objectives, a thorough "Literature Review" is provided to contextualize this work within existing studies. The "Methodology" section details the dataset preparation, model selection, training process, and performance evaluation framework, including metrics and explainability techniques. The "Results" section presents the comparative performance analysis of eight deep learning models across multiple metrics, supported by visualizations and performance summaries. In the "Discussion", the findings are critically analyzed to highlight the strengths, limitations, and practical implications of the tested architectures. Finally, the "Conclusion" summarizes the key outcomes, contributions, and future research directions aimed at enhancing automated foul detection systems for professional football officiating.

### Literature review

Table 1 offers a comparison of current research employing DL methods on sophisticated automatic foul detection and football video analysis. To enhance player tracking, foul recognition, and decision-making assistance in football matches, every research combines DL architectures, CV techniques, and data-driven methodologies in varying ways.

**Table 1:** Comparative review of DL techniques for automated foul detection and football video analysis.

References	Year	Technique Used	Study Description	Major Findings
^15^	2025	Hybrid DL Model (Object Tracking + Motion Analysis), Spatiotemporal Feature Extraction.	Suggests a CV and DL Artificial Intelligence (AI) system to instantly identify offside and fouls.	In actual match conditions, achieved 99.85% accuracy for offside identification and 98.56% for foul detection, therefore proving great dependability.
^16^	2025	VAR-YOLOv8s, MPDIoU, Residual Local Feature Network (RLFN), VARS Module, IoT.	Presents an IoT-enabled VAR-YOLOv8s model employing sensor and video data for real-time foul detection.	On SoccerNet, achieved IoU@0.5 of 80.5 and mAP@0.5 of 31.0, therefore indicating a smart referee system potential.
^17^	2024	YOLO, 3D-CNN, CNN, Faster R-CNN, LSTM, BLSTM, CV.	Tracks ball movement and team possessions using combination of object detection and DL.	Hybrid models improve tracking accuracy and offer deeper game insights.
^18^	2024	YOLOv8s, Global Attention Mechanism (GAM), P2 Detection Head, MPDIoU Loss Function.	Creates a better YOLOv8s model for football automated referee gesture detection.	Achieved 89.3% precision, outperforming standard YOLOv8s by up to 5.4% in key metrics.
^19^	2024	LAMP Network, Vue Framework, Canonical Correlation Analysis, SVM.	Creates a DL and data analysis intelligent system for football motion identification.	combines depth and skeleton elements to increase real-time feedback and motion recognition accuracy for training.
^10^	2024	DL, Multi-Object Tracking, Spatiotemporal Action Recognition.	Creates a system using broadcast footage to find players engaged in infractions, differentiate subjects (offers) from objects (victims).	Even with low-resolution footage, achieves great accuracy in spotting bad players and reasonable accuracy in separating offenders from victims.
^20^	2023	Deep Reinforcement Learning, Action Valuation, Event & Tracking Data Analysis.	Based on match statistics, uses deep reinforcement learning to find best actions for attacking and defensive players.	Defensive players should modify foul, clearing, and ball-out tactics depending on field position; offensive players should shoot more long-distance shots.
^21^	2023	Hybrid CNN + GCN, Data Augmentation, Multi-class Cross-Entropy Loss.	Creates a DL method combining CNN and GCN to categorize football player activity from images and video.	Achieved 97.4% accuracy, outperforming benchmarks in classifying 17 football activities using fused visual and pose data.
^22^	2021	Customized Detection Model, Spatial & Bounding Box Filters, Player Number Recognition.	Creates a DL system utilizing custom filters and t-shirt numbers to find, track, and recognize players.	achieves enhanced player recognition accuracy and high confidence player tracking with less identity swaps.

## Methodology

### Dataset presentation

Divided into two main classes—Foul and Not Foul—the 7000 images used in this study are from actual football match video. Each Foul scenario was kept in a different subfolder in each of the many folders the dataset was arranged into, while all Not Foul images were combined into one Not Foul folder. Preprocessing and class-wise parsing made possible by this hierarchical structure.

Three subsets—70% (4900 images) for training, 20% (1400 images) for validation, and 10% (700 images) for testing—were formed from the dataset. Aiming to contain 350 Foul and 350 Not Foul images, a balancing technique was used during test set construction; nevertheless, class distribution restrictions prevented always flawless achievement of this balance. Table 2 sums the last counts for every subgroup.

**Fig. 1:**
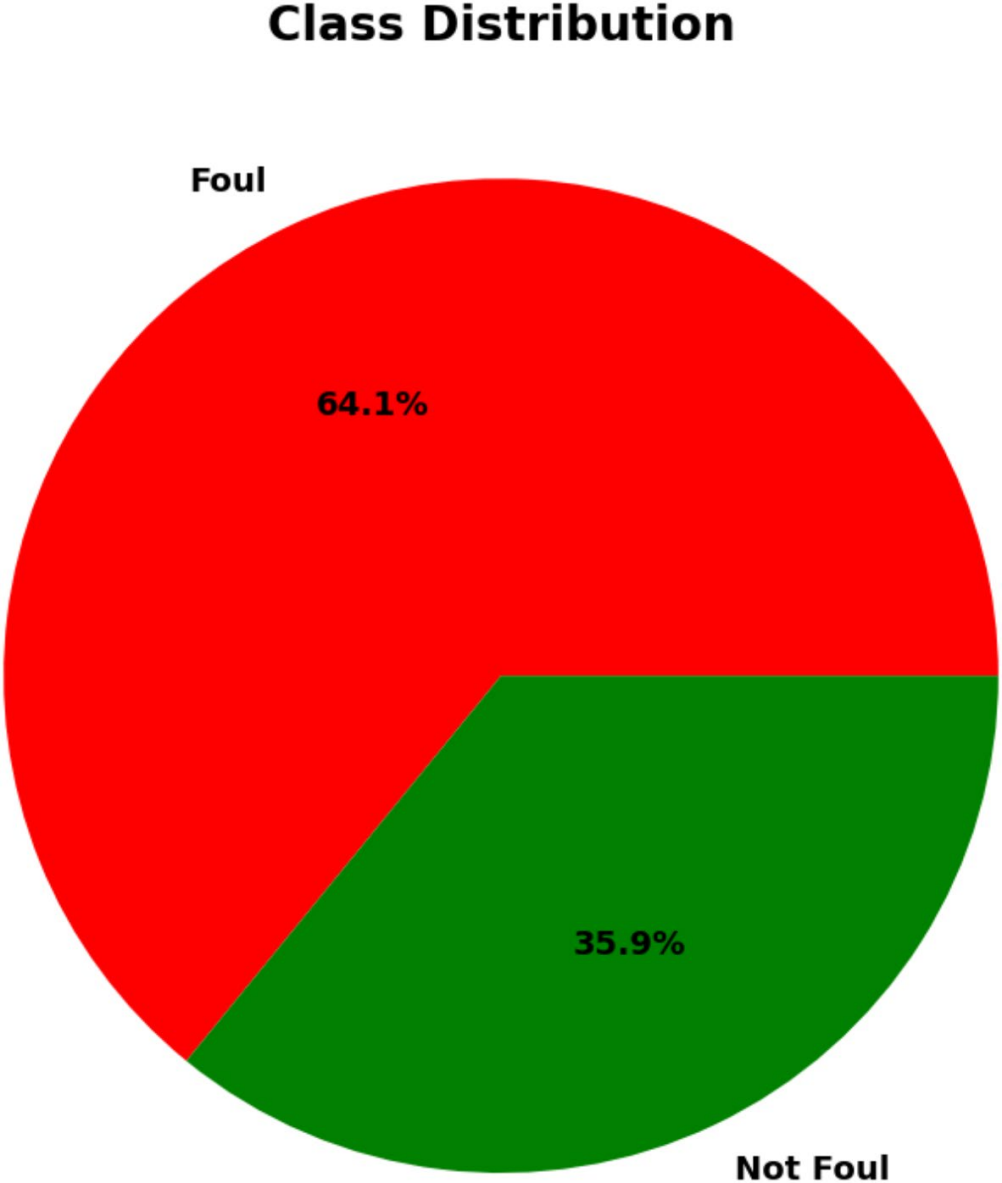
Class distribution in the dataset.

The class distribution overall showed that although 35.9% (2513 photos) were classed as Not Foul, 64.1% (4487 images) belonged to the Foul class. Figure 1 shows this distribution by pie chart of the class proportions.

The variations in annotation availability between the two classes define the dataset in one important way. foul images accompany dedicated (_annotations.csv) files with comprehensive object-level annotations for entities including the fouling player, the victim player, and the foul action zone itself. Every entry in these annotation files records the file name, image dimensions (width and height), class label, bounding box coordinates (x_min_, y_min_, x_max_, y_max_), so localizing the relevant areas for purposes of training and interpretability. On the other hand, Not Foul images are devoid of annotation files, since these images depict negative samples where none of the actions of interest take place. Thus, instead of using any region defined by a bounding box in training and evaluation for Not Foul images, the whole frame is used.

The region-based images associated with some performance annotations for Foul cases and full-frame images for Not Foul cases embody the real-world challenge in implementing automated foul-detection system in football, where specific fouling actions should be discriminated from general play.

**Table 2:** Dataset summary and distribution.

Dataset Split	Foul Samples	Not Foul Samples	Total Samples
Training Set	3283	1617	4900
Validation Set	937	463	1400
Test Set	350 (Actually 251)	350 (Actually 449)	700
Total	4487 (Actually 4570)	2513 (Actually 2430)	**7000**

### Methodological framework

In this study develops an automated foul detection system in football, based on a comparative evaluation of state-of-the-art DL architectures including EfficientNetV2, ResNet50, VGG16, Xception, InceptionV3, MobileNetV2, InceptionResNetV2, and DenseNet121. The methodology has a pipeline consisting of dataset preparation, model development, training along with explainability integration, and comparative performance evaluation.

#### Dataset collection and preprocessing

Images divided into two classes—Foul and Not Foul—were gathered for a complete soccer foul detection dataset. The dataset was split into several subfolders, each reflecting a different foul scenario (such as Foul_1, Foul_2), where each folder had images together with matching bounding box annotations found in (_annotations.csv) files. These annotations enabled the particular areas within images where fouls happened to be localized.

Custom Python scripts were created to handle the dataset by parsing the images files and related annotations. The bounding boxes were taken out and cropped and isolated the pertinent foul areas for images labeled as such. On non-foul images, on the other hand, the whole image was utilized without cropping. To guarantee consistency and compatibility throughout all DL architectures applied in this work, all photos were shrunk to a standard dimension of 224 × 224 pixels.

The dataset was then divided at random into three subsets: 70% of the images were used as training data, 20% were used as validation data, and the remaining 10% are reserved for testing, all using stratified sampling to ensure even distribution of the classes in each subset. Moreover, the test dataset was balanced to ensure equal numbers of foul and non-foul images (with a target of 350 images per class, subjected to the availability of adequate data per class).

The second step is to visualize the dataset, for example, class-wise histograms, pie charts, and previewing few samples of images with bounding boxes for the foul ones, so that the study could get a better insight of the dataset and check whether the dataset is good enough for training the model or not. These visualizations gave an overall idea about classes distribution and also allowed to visualize any possible bias or inconsistency in the dataset before setting off to training.

#### Model architectures and setup

Images divided into two classes—Foul and Not Foul—were gathered for a complete soccer foul detection dataset. The dataset was split into several subfolders, each reflecting a different foul scenario (such as Foul_1, Foul_2), where each folder had images together with matching bounding box annotations generated in (*_annotations.csv*). These annotations enabled the particular areas within images where fouls happened to be localized.

This research assesses and contrasts eight pre-trained DL architectures for the purpose of automatic foul identification in football. The chosen models—EfficientNetV2, ResNet50, VGG16, Xception, InceptionV3, MobileNetV2, InceptionResNetV2, and DenseNet121—are well-established CNNs. From scalable efficiency in EfficientNetV2 to lightweight deployment adaptability in MobileNetV2 and densely coupled feature propagation in DenseNet121, every architecture presents unique design concepts and characteristics. Table 3 summarizes these designs together with their primary differentiating characteristics and input sizes.

Their convolutional feature extraction layers were frozen in order to modify these pre-trained models for binary foul classification, thereby conserving the learnt representations from ImageNet. Comprising a Global Average Pooling layer, a fully connected dense layer with 1024 units and Rectified Linear Unit (ReLU) activation, and a last sigmoid output layer to forecast the chance of a foul on top of the frozen basis, a custom classification head was added. Using a binary crossentropy loss function, accuracy as the main evaluation parameter, and a 0.0001 learning rate Adam optimizer, all models were constructed.

**Table 3:** Summary of pre-trained models used in foul detection.

Model	Input Size	Key Strength
EfficientNetV2	224 × 224	Scalable & efficient
ResNet50	224 × 224	Residual learning
VGG16	224 × 224	Simplicity & baseline
Xception	224 × 224	Depthwise separable convolutions
InceptionV3	224 × 224	Multi-scale feature extraction
MobileNetV2	224 × 224	Lightweight for mobile and edge devices
InceptionResNetV2	224 × 224	Hybrid architecture (Inception + Residual)
DenseNet121	224 × 224	Dense connectivity between layers

#### Training process

These datasets were processed using (tf.data pipelines) to make data loading and augmentation easier. Images were loaded and preprocessed on-the-fly during training, and foul images were optionally cropped to the annotated bounding boxes (if available). The shuffling prevents any sort of ordering bias and batching with (batch_size = 32), and within the start of the training, prefetching was also used to optimize so that data flow is also in the same pace as the training process – this makes sure that there is no latency during training.

Several callbacks were used to improve training resilience and efficiency. If the validation loss did not improve for ten straight epochs, early stopping was utilized to end training and hence avoid overfitting. Furthermore, included was a technique for model check pointing to store the architecture with lowest validation loss. A GradCAM + + visualization callback was also included into the explainability integration to create visual heatmaps following every epoch, so emphasizing the image areas impacting the decisions of the model. Though early stopping usually ended training between 50 and 120 epochs, depending on the model’s convergence behavior and stability across various topologies, each model was trained for a maximum of 300 epochs.

#### Comparative analysis of DL architectures

This section provides a comparative study of the eight DL architectures used in this study for automated football foul detection. Key characteristics, approaches to handling features, strengths, and limitations for each model are summarized in Table 4, which provides a holistic comparison of models in relation to this task. The architectures used ranges from traditional convolutional networks such as regular VGG16 to modern, efficiency focused designs like EfficientNetV2 and MobileNetV2, as well as advanced hybrid architectures such as InceptionResNetV2.

Different models have differing complexities; for example, MobileNetV2 was created specifically with lightweight applications in mind, whereas other models like InceptionResNetV2 and ResNet50 use deeper designs and advanced residual or inception modules which add to their complexity. Feature-wise, architectures like InceptionV3 use parallel convolutional paths to capture multi-scale features, and DenseNet121 reuses features by providing dense connectivity across layers. These design decisions influence performance, but also the ability of the models to adapt behavior to new data, as well to remain robust to changes to the conditions.

As indicated in Table 4, MobileNetV2 achieves superior computational efficiency, rendering it particularly relevant in real-time applications like VAR systems. While approaches such as DenseNet121 and InceptionResNetV2 have better feature learning capacity and generalization potential, and can be regarded as potential approaches for high-performance systems for foul detection. But these advantages come at the cost of limitations, including longer training times, higher propensity for overfitting in the case of deeper models and loss in fine-grained detail detection in some of the lightweight architectures. This comparison highlights the need for thoughtful model selection based on the desired trade-offs between these performance domains, especially in light of potential deployments in existing officiating technologies like VAR.

**Table 4:** Comparative analysis of DL algorithms for automated foul detection in football, highlighting architectural characteristics, feature handling approaches, strengths, and limitations.

Algorithm	Type	Complexity	Feature Handling	Strengths	Limitations
EfficientNetV2^23^	CNN	Moderate	Compound scaling across depth, width, resolution	Efficient with good accuracy	Struggles with fine-grained fouls in fast scenes
ResNet50^24^	CNN	High	Residual connections enhance DL	Strong feature extraction, good for transfer learning	Prone to overfitting, slower training
VGG16^25^	CNN	High	Sequential deep layers	Simple design, strong feature extraction	Large, slower inference, overfits without augmentation
Xception^26^	Depthwise CNN	High	Separates spatial and channel-wise learning	Efficient, reduced computation	More complex training, struggles on small datasets
InceptionV3^27^	Inception CNN	High	Parallel multi-scale feature extraction	Captures multi-scale features well	Complex architecture, moderate inference
MobileNetV2^28^	Lightweight CNN	Low	Depthwise convolutions, inverted residuals	Extremely lightweight, mobile-friendly	Lower accuracy on complex fouls
InceptionResNetV2^29^	Hybrid CNN	Very High	Residual + multi-scale feature extraction	High accuracy, balanced feature learning	Very complex, slower training and inference
DenseNet121^30^	Dense CNN	High	Maximizes feature reuse across layers	Strong feature propagation, efficient	Slightly higher memory cost

#### Summary of the methodological workflow

**Fig. 2:**
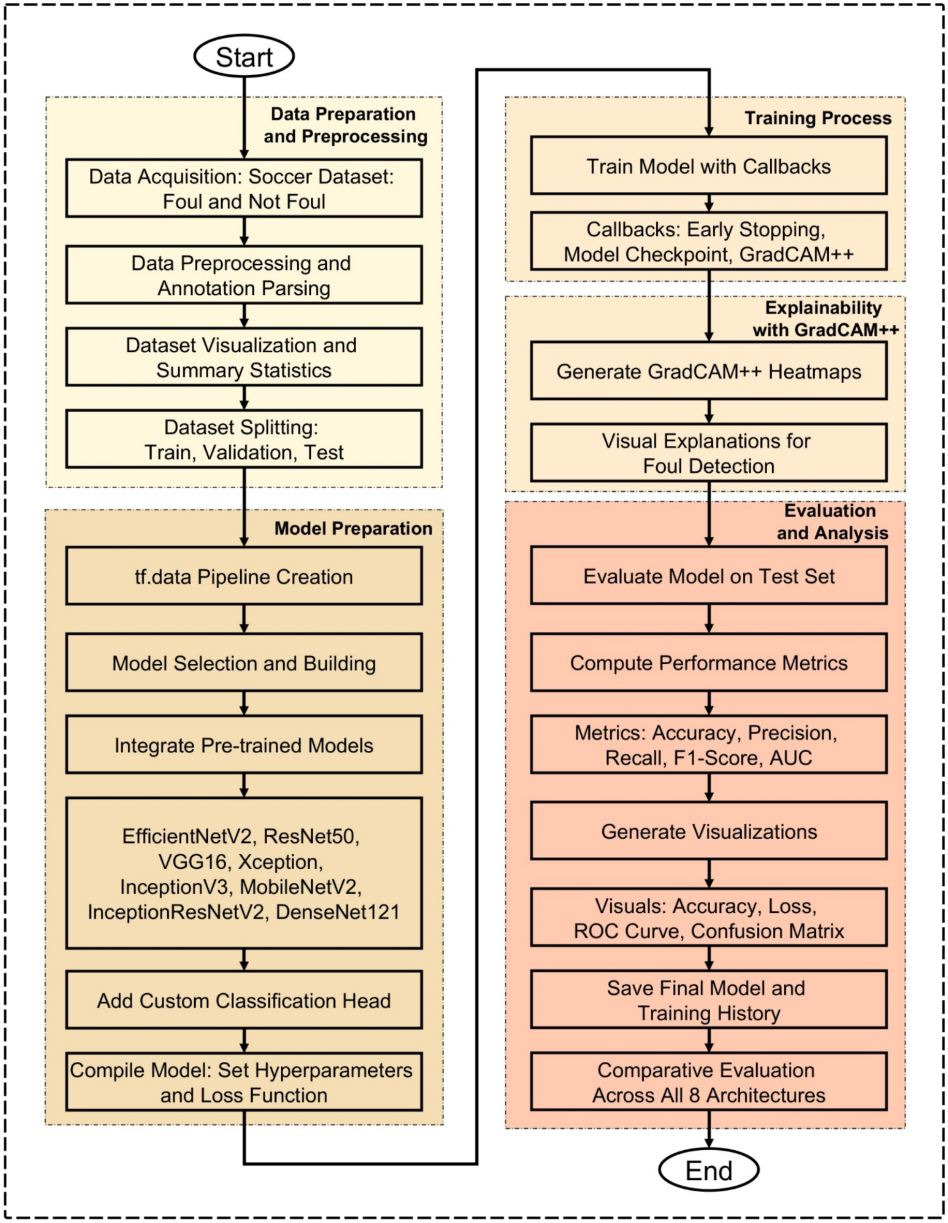
Flowchart for advanced automated foul detection in football using DL architectures.

Figure 2 shows the general methodological flow of this work, which shows a well-organized pipeline spanning all important phases, from data preparation to last comparative evaluation. Data collecting and preprocessing start the process; the soccer foul dataset is gathered, annotated, visualized, and separated into training, validation, and test sets. After that, the model preparation stage consists in the development of (tf.data pipelines) for effective data loading, the choice and integration of eight pre-trained DL models, and the inclusion of a custom classification head catered for binary foul classification. Each model is then compiled with a specified optimizer, loss function, and evaluation metric and trained with many optimizations, including early stopping to reduce overfitting, model check pointing to save each architecture’s best-performing model, and usage of GradCAM + + to produce heatmaps indicating regions of the original image that contributed to the model’s predictions. This is done by generating visual explanations at the conclusion of each epoch, and provides a useful increase in the interpretability of the system. Ultimately, during the evaluation and analysis phase, all models are evaluated on the balanced test set with detailed performance metrics — including accuracy, precision, recall, F1 score, and AUC — along with visual diagnostics (accuracy and loss curves, Receiver Operating Characteristic (ROC) curves, and confusion matrices). The process ends with a comparison of all eight architectures to get the best model for automatic foul detection. As shown in Fig. 2, this methodical and open structure guarantees that the suggested strategy is both interpretable and scientifically strong.

### Parameters description

Training and evaluating the eight DL architectures for automated foul detection in football required defining a list of key parameters that were used to control the data preprocessing, the model training, and evaluation processes. The adopters of these attributes were fixed across all models because they were determined based on the best practices available in deep learning.

Across all models, the input image size was set at 224 × 224 pixels to match the pre-trained architectures’ input needs and preserve data pipeline homogeneity. Setting a batch size of thirty-two images for training and validation balanced memory efficiency with training stability. By scaling pixel values, every image was brought into line with pre-trained model expectations—a [0, 1] range.

All the models were compiled with Adam optimizer with a learning rate of 0.0001, which offered a nice trade-off between speed of convergence and stability. For the binary classification (foul vs. not foul), the binary cross-entropy loss function was used to train the models, and accuracy was the main performance metric used during training. To improve training speed, the study enabled early stopping with a patience value of 10 epochs, or stopping training if the validation loss did not improve for 10 consecutive epochs.

Every architecture’s lowest validation loss model was automatically saved using a model check pointing system. At the end of every training session, a GradCAM + + callback was also included to create visual explanations for a subset of photos, therefore offering interpretable analysis of how each model found fouls. Table 5 shows a list of the main values applied in all models.

**Table 5:** Summary of key parameters used in model training and evaluation.

Parameter	Value / Description
Input Image Size	224 × 224 pixels
Batch Size	32
Image Normalization	Rescaled to [0, 1]
Optimizer	Adam
Learning Rate	0.0001
Loss Function	Binary Crossentropy
Primary Metric	Accuracy
Secondary Metrics	Precision, Recall, F1 Score, AUC
Maximum Epochs	300
Early Stopping Patience	10 epochs
Model Checkpointing	Save model with lowest validation loss
Explainability Technique	GradCAM++
GradCAM + + Samples per Epoch	5 per training, validation, and test sets
Test set balance	350 Foul, 350 Not Foul (where feasible)
Train/Validation/Test Split	70% / 20% / 10%
Data Pipeline Framework	TensorFlow tf.data

### Evaluation metrics

In this research, the study adopts a set of established evaluation metrics, which offer both overall and per-class analysis, to rigorously assess the performance of the proposed automated foul detection system. These are important metrics for general accuracy as well as capturing how well the models are discerning foul events from non-foul events. The metrics used are the Accuracy, Precision, Recall, F1 Score, and the Area Under the Receiver Operating Characteristic Curve (AUC-ROC).

Accuracy gives a general picture of model performance by indicating the percentage of properly identified events over both classes. The study computes it with Eq. (1). But accuracy by itself might be deceptive in normal match video as foul occurrences are less common than non-foul events, particularly if the model leans towards the majority class.

Precision emphasizes the validity of the negative predictions, therefore reflecting the percentage of the cases categorized as fouls that were truly fouls. Maintaining the dependability of automated analysis depends on less false positive foul detection, so a better accuracy is necessary. Calculating precision with Eq. (2)

Recall, often known as sensitivity, gauges the accuracy of the model in spotting real fouls, therefore capturing the percentage of true fouls accurately found. Excellent recall guarantees that the system reduces missed foul observations. Recall has a formula shown in Eq. (3).

The F1 Score is utilized to get a fair evaluation of accuracy and recall as both of these measures usually show a trade-off. Emphasizing situations in which both metrics are equally essential, the F1 Score is the harmonic mean of accuracy and recall. The F1 Score is defined by Eq. (4).

Finally, the model’s ability to discriminate between foul vs. non-foul events across a range of decision thresholds is evaluated using the AUC-ROC statistic. With TPR defined by Eq. (5) and FPR defined by Eq. (6) respectively, the ROC Curve plots the True Positive Rate (TPR) against the False Positive Rate (FPR) at different classification thresholds. The AUC calculates the area under the curve; the higher the better the model’s ability to distinguish between classes.

Taken together, these metrics allow for a holistic and balanced assessment of the efficacy of the model, allowing both a fair comparison between the eight architectures tested, and an unfortunate indication of the system’s feasibility for uses for transcendental settings for physical foul detection.

1 $$\:Accuracy=\frac{TP+TN}{TP+TN+FP+FN}$$

2 $$\:Precision=\frac{TP}{TP+FP}$$

3 $$\:Recall=\frac{TP}{TP+FN}$$

4 $$\:F1-Score=\frac{2\times\:Precision\times\:Recall}{Precision+Recall}$$

5 $$\:TPR=\frac{TP}{TP+FN}$$

6 $$\:FPR=\frac{FP}{FP+FN}$$

## Results

This section discusses the entire evaluation of the DL architectures aimed at automatically detecting fouls in football. The performance of various deep learning models, like EfficientNetV2, ResNet50, VGG16, Xception, InceptionV3, MobileNetV2, InceptionResNetV2, and DenseNet121, is evaluated on various assessment metrics. These include training and validation performance, test accuracy and loss, as well as precision, recall, F1-score, and AUC metrics. Additionally, visual representations of training and validation accuracy and loss, ROC curves, confusion matrices, and comparative performance metrics are provided to facilitate a more profound understanding of the strengths and limitations of each model.

### Training and validation performance

Table 6 summarizes the training and validation performance of the assessed models, offering insights into their capacity to generalize to novel data. The findings demonstrate that DenseNet121 and MobileNetV2 attained a flawless training accuracy of 100%, underscoring their remarkable ability to assimilate information from the training data. Nevertheless, higher training accuracy does not inherently ensure robust generalization. Of all the models, DenseNet121 had the greatest validation accuracy at 0.9764, followed by MobileNetV2 at 0.9686 and VGG16 at 0.9600. This indicates that these designs excelled on the training dataset and preserved their prediction capability when evaluated on novel data. Regarding training loss, DenseNet121 achieved the lowest value (0.006), succeeded by MobileNetV2 (0.0086) and InceptionV3 (0.0236), indicating negligible prediction mistakes during training. The validation loss findings further substantiate the exceptional performance of DenseNet121, which attained the lowest validation loss (0.0621), signifying robust generalization ability. Conversely, ResNet50 exhibited a validation loss of 0.2098, indicating a much greater extent of overfitting compared to the leading models. The results indicate that DenseNet121, MobileNetV2, and InceptionV3 demonstrate enhanced training and validation performance, positioning them as formidable contenders for automated foul identification in football.

**Table 6:** Training and validation performance of DL models for automated foul detection in football.

Model	Epoch Number	Training Accuracy	Training Loss	Validation Accuracy	Validation Loss
EfficientNetV2	44	0.6609	0.5826	0.6836	0.5414
ResNet50	134	0.953	0.1464	0.9171	0.2098
VGG16	78	0.9912	0.0348	0.9600	0.1056
Xception	16	0.9961	0.0264	0.9471	0.1206
InceptionV3	18	0.9961	0.0236	0.9536	0.1313
MobileNetV2	17	1.0000	0.0086	0.9686	0.0817
InceptionResNetV2	37	0.9961	0.0191	0.9629	0.0828
DenseNet121	30	1.0000	0.006	0.9764>	0.0621

### Test performance evaluation

Table 7 presents the models test performance on the important assessment metrics test loss, accuracy, precision, recall, F1-score, and AUC. In summary, the results show that the best test accuracy of 0.8757 was achieved by InceptionResNetV2 which outran all others in this respect, whereas DenseNet121 and MobileNetV2 closely followed behind with an accuracy of 0.8686. This illustrates the capacity of these models to generalize well to previously unobserved test data. Precision, reflecting the ratio of accurate positive predictions, was best for DenseNet121 (0.9786), demonstrating its efficacy in reducing false positives. In terms of recall, InceptionResNetV2 achieved the highest score (0.8396), indicating its superior efficacy in detecting pertinent positive cases. InceptionResNetV2 (0.8966) led the F1-score, which balances accuracy and recall; followed by DenseNet121 (0.8881) and MobileNetV2 (0.8889), hence underlining the durability of these designs. DenseNet121 (0.9641) has the highest area under the curve (AUC) score followed closely by InceptionResNetV2 (0.962) and MobileNetV2 (0.961), which evaluates the general classification efficacy of the model. With InceptionResNetV2 excelling in recall and F1-score, DenseNet121 exceeded in accuracy and AUC, while MobileNetV2 displayed better test performance across multiple assessment criteria.

**Table 7:** Performance comparison of DL models for automated foul detection in football.

Model	Test Loss	Test Accuracy	Precision	Recall	F1-Score	AUC
EfficientNetV2	0.5202	0.6900	0.7458	0.7840	0.7644	0.7668
ResNet50	0.457	0.8429	0.9335	0.8129	0.869	0.9153
VGG16	0.6222	0.8400	0.9616	0.7817	0.8624	0.9383
Xception	0.4668	0.8614	0.9536	0.8241	0.8841	0.9413
InceptionV3	0.417	0.8614	0.9467	0.8307	0.8849	0.9526
MobileNetV2	0.4392	0.8686	0.971	0.8196	0.8889	0.9601
InceptionResNetV2	0.3986	0.8757	0.9617	0.8396	0.8966	0.9622
DenseNet121	0.4772	0.8686	0.9786	0.8129	0.8881	0.9641

### Visual performance analysis

Multiple visual representations were produced to fully examine the performance trends of the evaluated DL models. These figures offer deeper insights on how each model performed during training and validation, together with comparative evaluations based on important criteria including accuracy, loss, AUC, and classification performance.

**Fig. 3:**
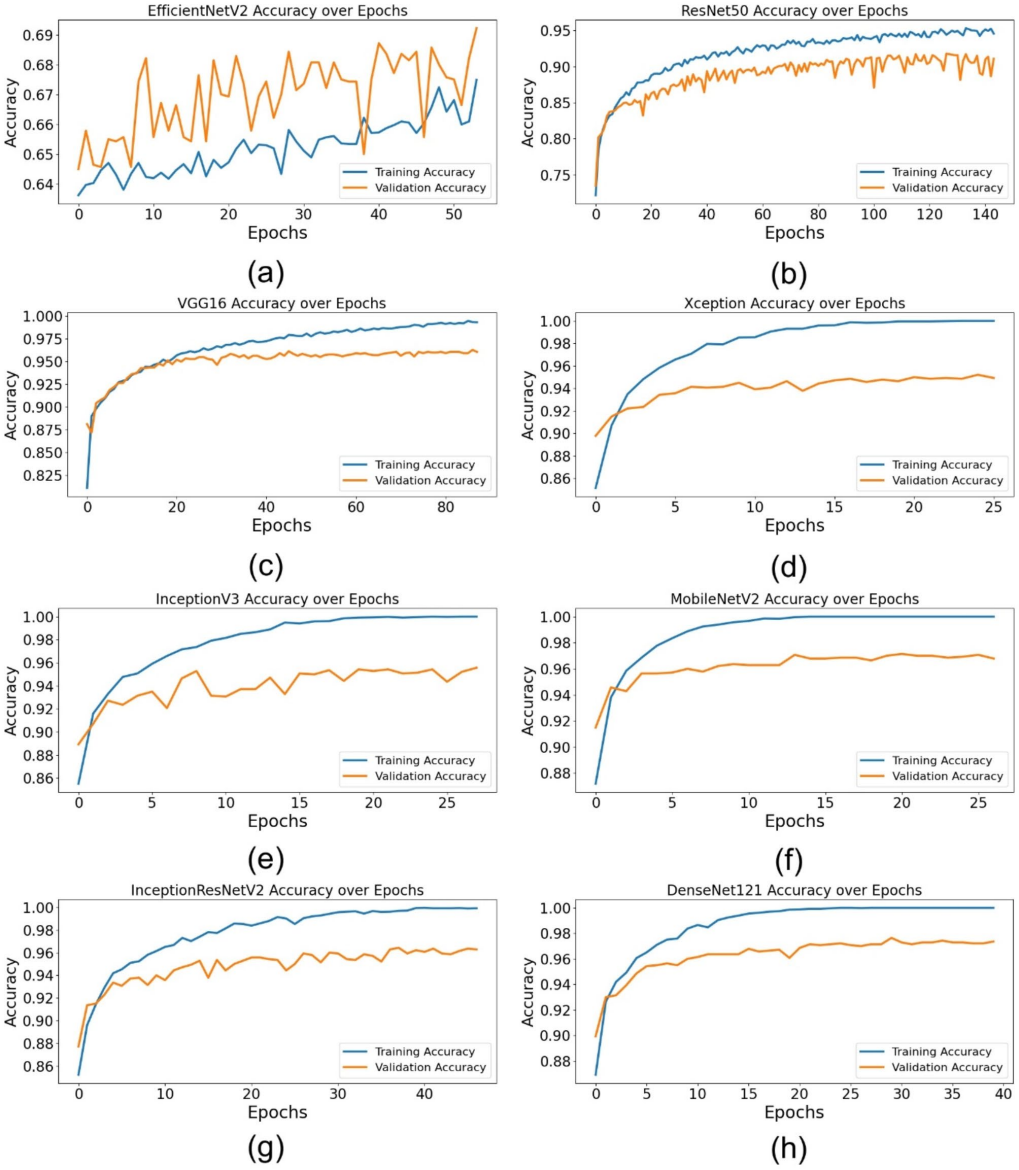
Training and validation accuracy of DL architectures for automated foul detection in football: (**a**) EfficientNetV2, (**b**) ResNet50, (**c**) VGG16, (**d**) Xception, (**e**) InceptionV3, (**f**) MobileNetV2, (**g**) InceptionResNetV2, (**h**) DenseNet121.

Figure 3 depicts the training and validation accuracy throughout epochs for all models. Figure 3(a) illustrates EfficientNetV2, Fig. 3(b) depicts ResNet50, Fig. 3(c) showcases VGG16, Fig. 3(d) exhibits Xception, Fig. 3(e) represents InceptionV3, Fig. 3(f) displays MobileNetV2, Fig. 3(g) relates to InceptionResNetV2, and Fig. 3(h) provides DenseNet121. The graphs indicate that DenseNet121, MobileNetV2, and InceptionResNetV2 attained the maximum validation accuracy, but EfficientNetV2 displayed significant swings, suggesting unstable learning.

**Fig. 4:**
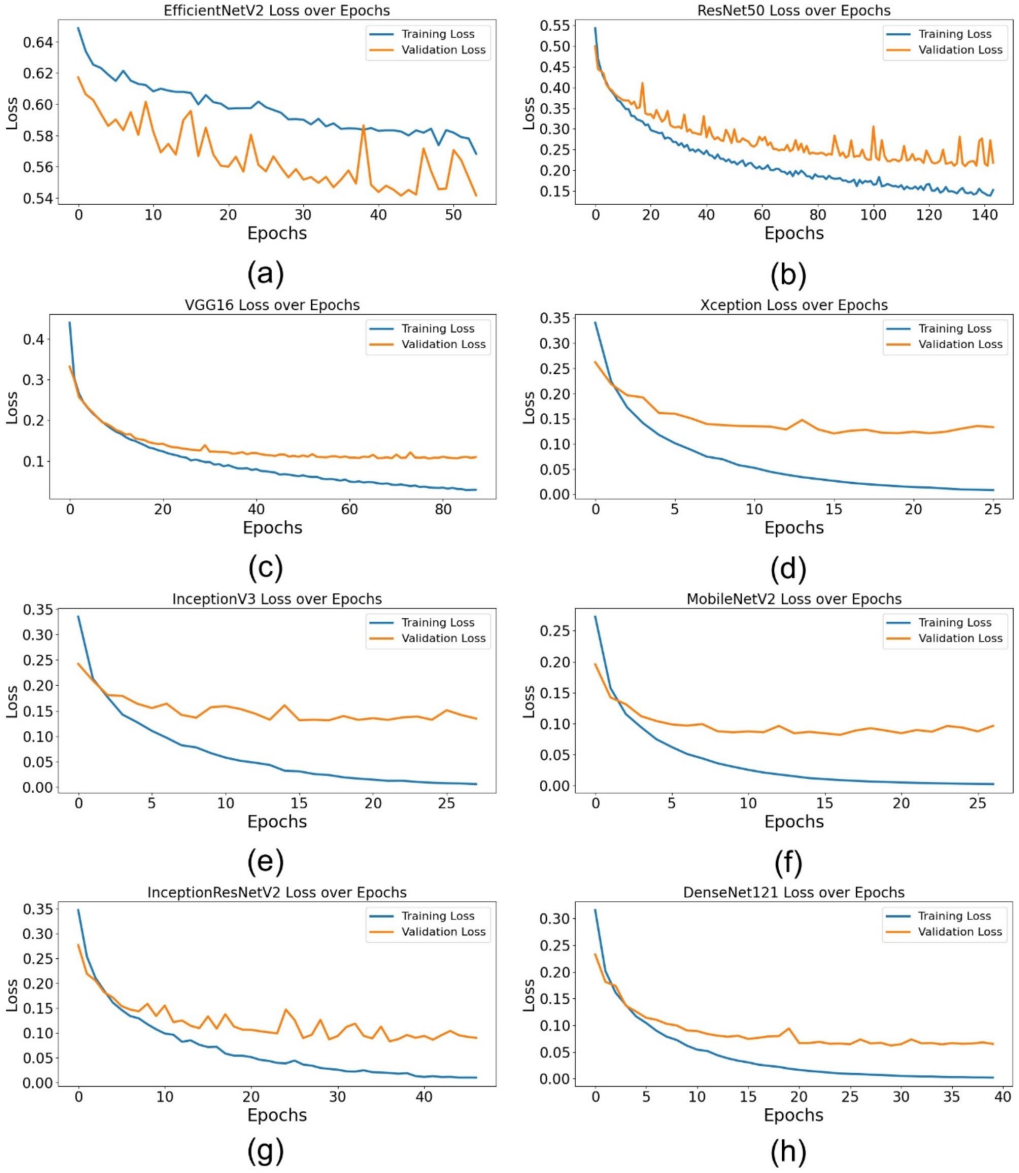
Training and validation loss of DL architectures for automated foul detection in football: (**a**) EfficientNetV2, (**b**) ResNet50, (**c**) VGG16, (**d**) Xception, (**e**) InceptionV3, (**f**) MobileNetV2, (**g**) InceptionResNetV2, (**h**) DenseNet121.

Figure 4 illustrates the training and validation loss throughout epochs for all models. Figure 4(a) represents EfficientNetV2, Fig. 4(b) denotes ResNet50, Fig. 4(c) illustrates VGG16, Fig. 4(d) depicts Xception, Fig. 4(e) showcases InceptionV3, Fig. 4(f) features MobileNetV2, Fig. 4(g) highlights InceptionResNetV2, and Fig. 4(h) presents DenseNet121. The findings indicate that DenseNet121 and MobileNetV2 had the lowest validation loss, reflecting enhanced generalization skills, but EfficientNetV2 experienced elevated and erratic validation loss, implying challenges in model convergence.

**Fig. 5:**
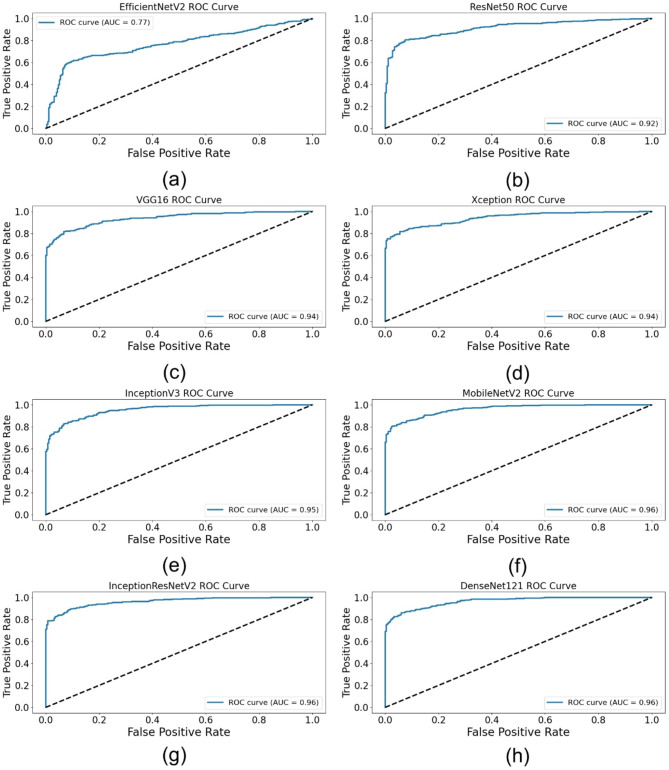
ROC curves of DL architectures for automated foul detection in football: (**a**) EfficientNetV2, (**b**) ResNet50, (**c**) VGG16, (**d**) Xception, (**e**) InceptionV3, (**f**) MobileNetV2, (**g**) InceptionResNetV2, (**h**) DenseNet121.

Figure 5 displays the ROC curves for each model, demonstrating the balance between the TPR and the FPR. Figure 5(a) illustrates EfficientNetV2, Fig. 5(b) depicts ResNet50, Fig. 5(c) showcases VGG16, Fig. 5(d) represents Xception, Fig. 5(e) features InceptionV3, Fig. 5(f) displays MobileNetV2, Fig. 5(g) highlights InceptionResNetV2, and Fig. 5(h) portrays DenseNet121. The findings demonstrate that DenseNet121, MobileNetV2, and InceptionResNetV2 attained the greatest AUC values, validating their exceptional classification efficacy.

**Fig. 6:**
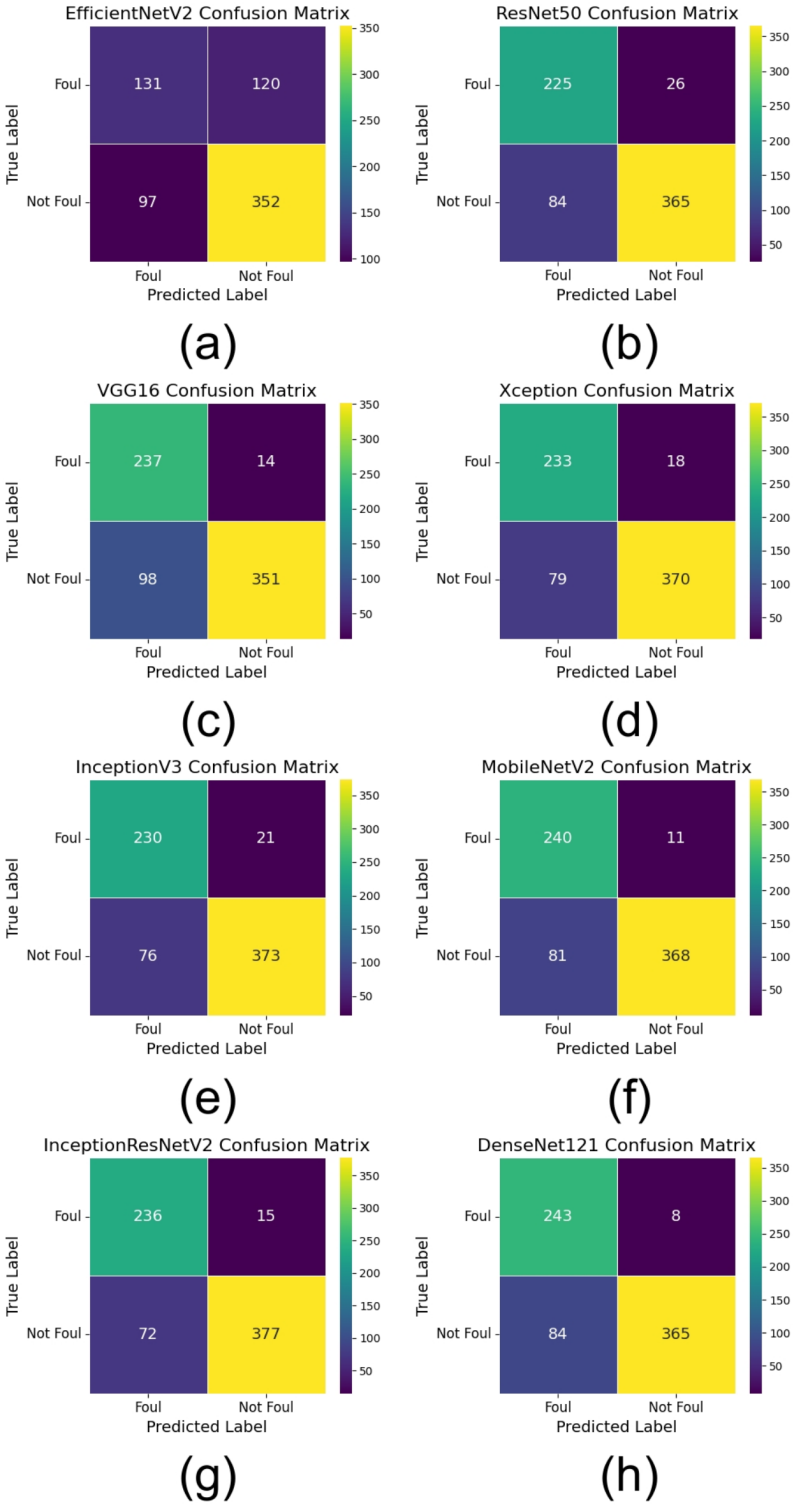
Confusion matrices of DL architectures for automated foul detection in football: (**a**) EfficientNetV2, (**b**) ResNet50, (**c**) VGG16, (**d**) Xception, (**e**) InceptionV3, (**f**) MobileNetV2, (**g**) InceptionResNetV2, (**h**) DenseNet121.

Figure 6 presents the confusion matrices for all models, illustrating their classification distributions to provide a more in-depth analysis of model performance. Figure 6(a) depicts EfficientNetV2, Fig. 6(b) illustrates ResNet50, Fig. 6(c) showcases VGG16, Fig. 6(d) represents Xception, Fig. 6(e) features InceptionV3, Fig. 6(f) displays MobileNetV2, Fig. 6(g) highlights InceptionResNetV2, and Fig. 6(h) portrays DenseNet121. The confusion matrices indicate that DenseNet121 and InceptionResNetV2 had the lowest misclassification rates, demonstrating robust differentiation between foul and non-foul situations.

**Fig. 7:**
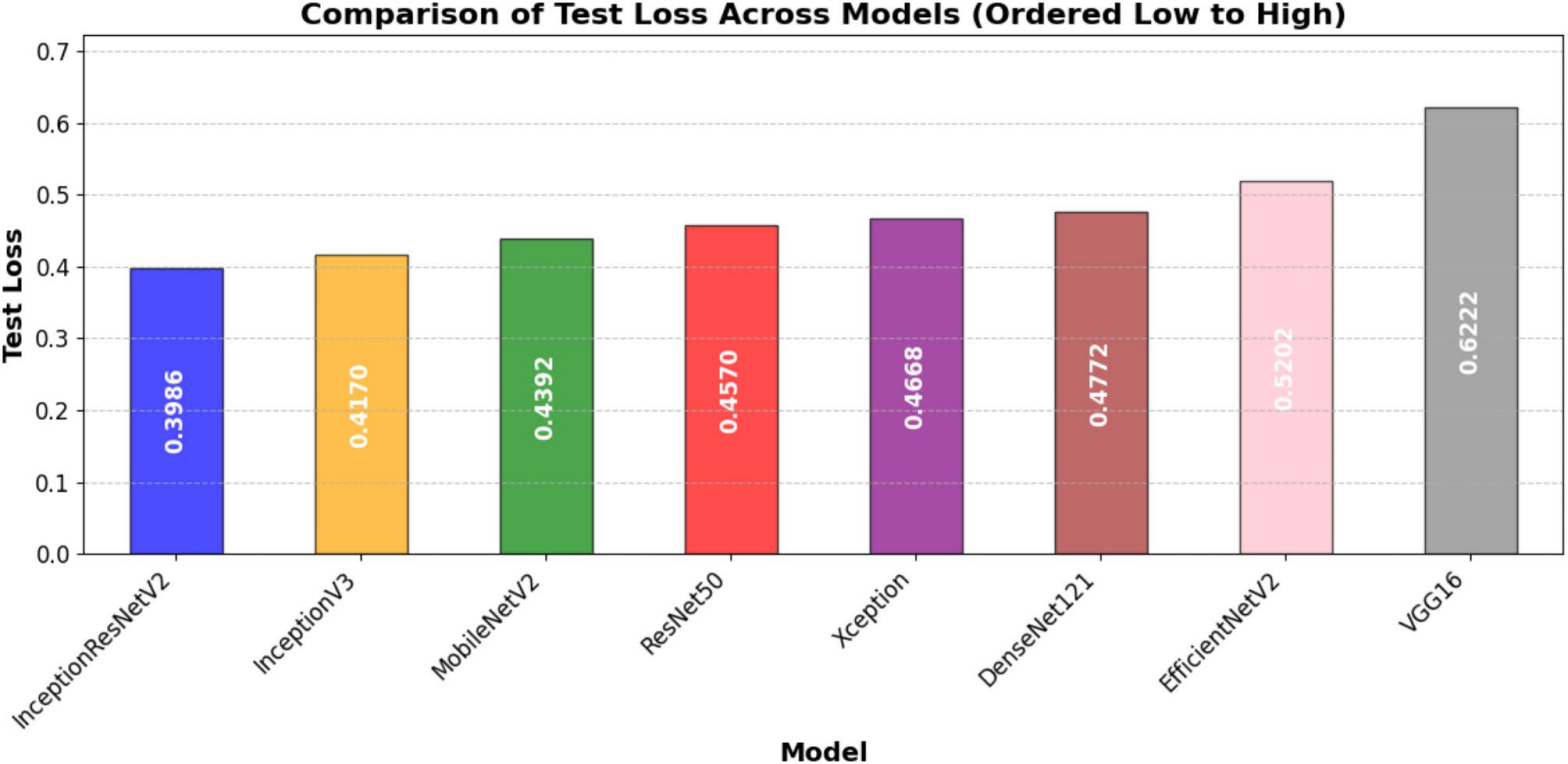
Ordered comparative analysis of test loss in DL models for automated football foul detection.

**Fig. 8:**
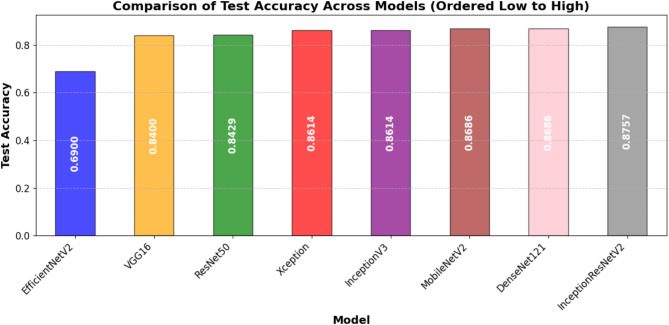
Ordered comparison of test accuracy in DL models for automated football foul detection.

**Fig. 9:**
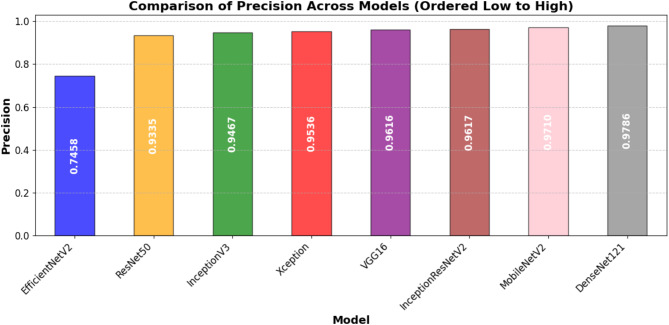
Ordered comparative analysis of precision in DL models for automated football foul detection.

**Fig. 10:**
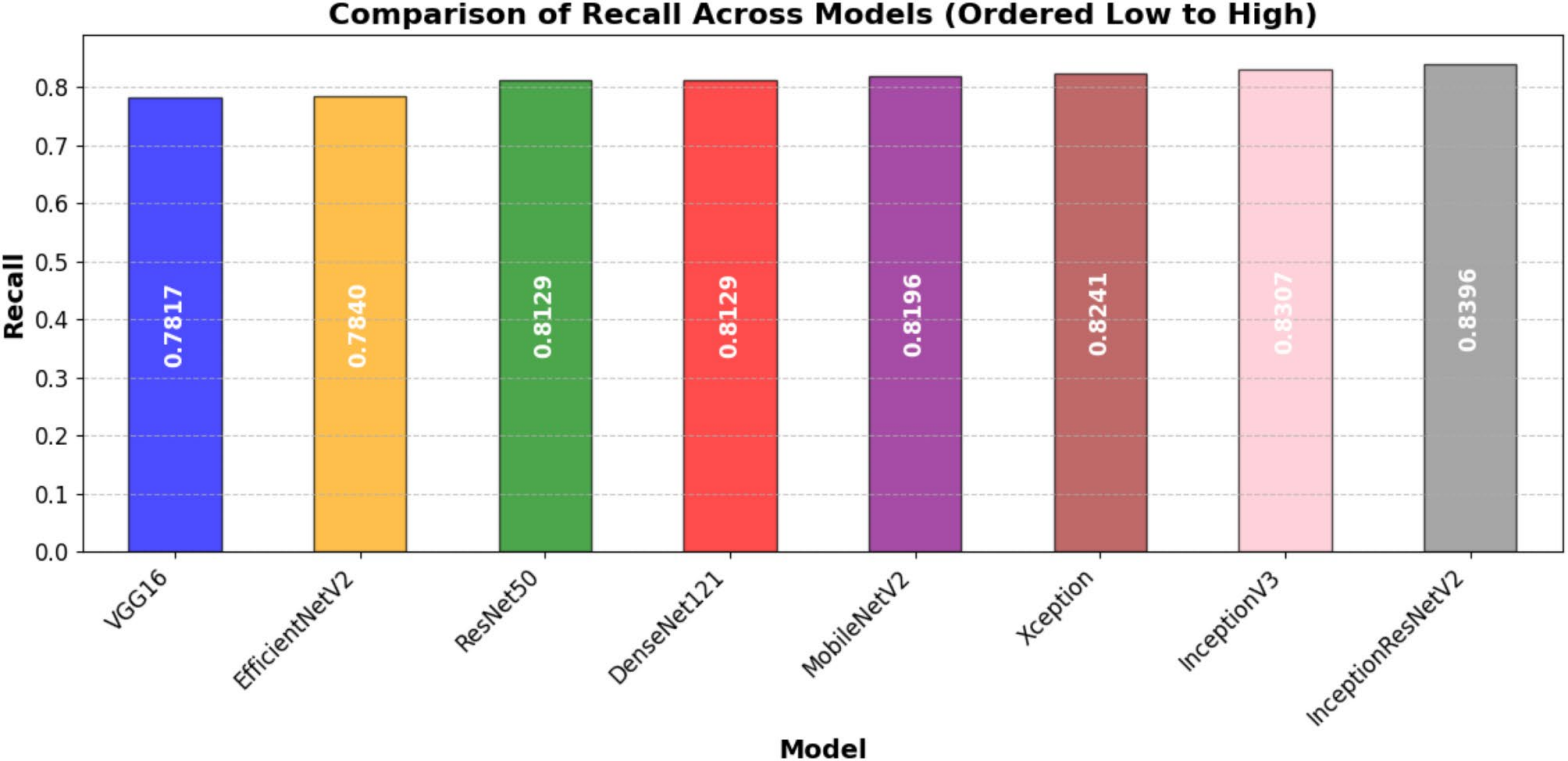
Ordered comparative analysis of recall in DL models for automated football foul detection.

**Fig. 11:**
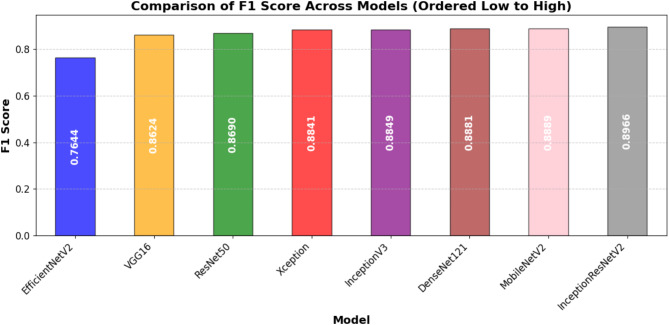
Ordered comparative analysis of F1-Score in DL models for automated football foul detection.

**Fig. 12:**
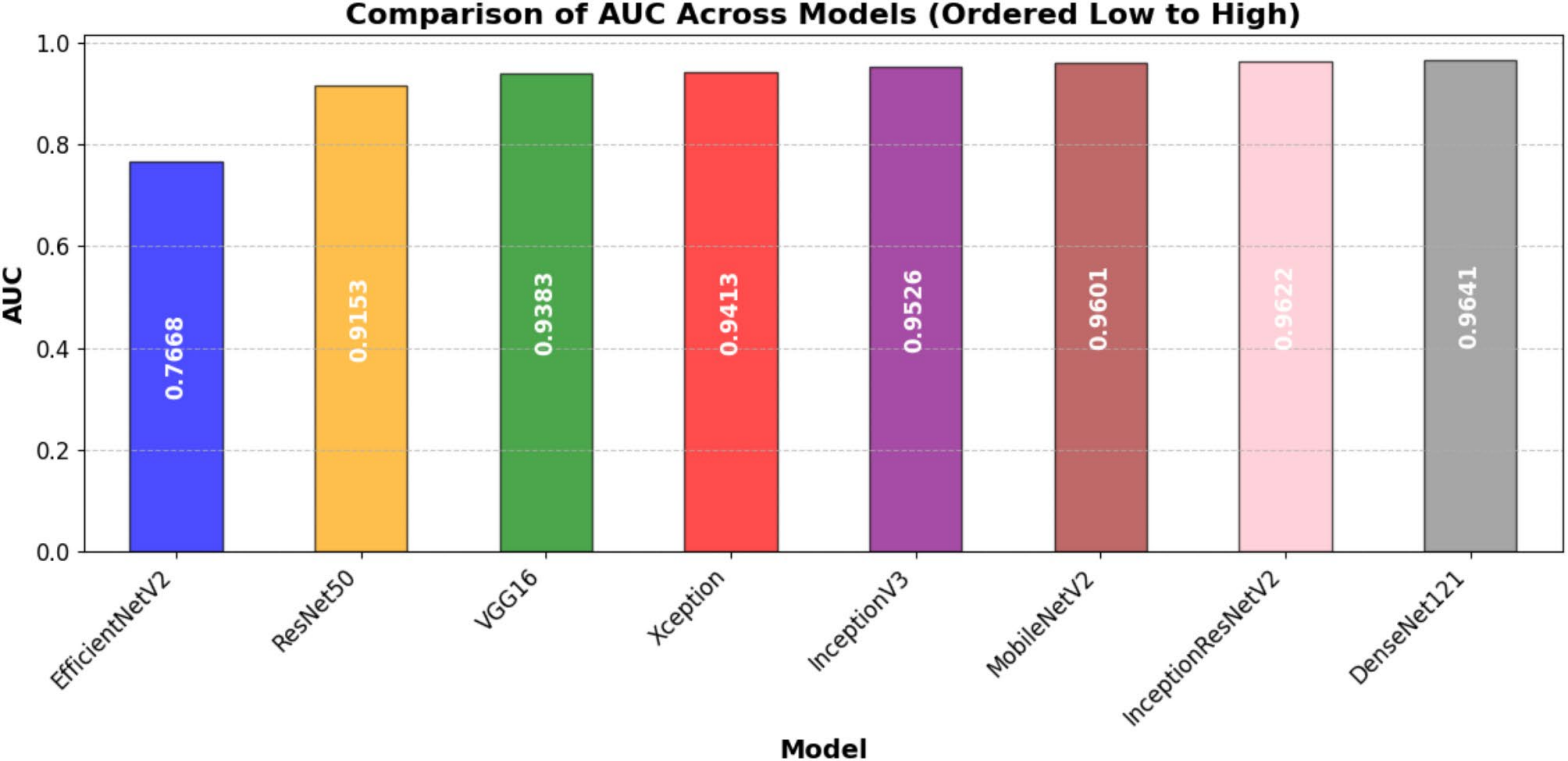
Ordered comparative analysis of AUC in DL models for automated football foul detection.

In addition to individual model marks, Figures from Figs. 7, 8, 9, 10, 11 and 12 provide relative analyses of all models. While InceptionResNetV2 achieved the lowest test loss, Fig. 7 provides a methodological comparison of test loss across models, ordered by test performance in prediction reducing error. With a ranked comparison of test accuracy, Fig. 8 shows that MobileNetV2, DenseNet121, and InceptionResNetV2 reached the highest degrees of accuracy. Figure 9 shows the ranked comparison of precision and names DenseNet121 as the model with the highest precision score. Figure 10 shows the methodical comparison of recall, where InceptionResNetV2 was very good at precisely identifying positive cases. InceptionResNetV2 exceeded all other models according to Fig. 11, a ranked comparison of the F1-score that strikes a mix between accuracy and recall. Figure 12 finally shows the methodical comparison of AUC values, thus verifying that DenseNet121 and InceptionResNetV2 achieved the better classification performance.

The early stopping mechanism was employed in all models (as covered in the training script), which was critical for optimized training and to avoid overfitting. Training was performed for a max of 300 epochs, with early stopping to halt training when validation performance no longer improved. In particular, the study applied an early stopping function to monitor validation loss and stop the training process whenever no further decrease was observed on validation loss in ten epochs — while restoring the model parameters based on the best-performing model on the validation dataset for generalization.

**Fig. 13:**
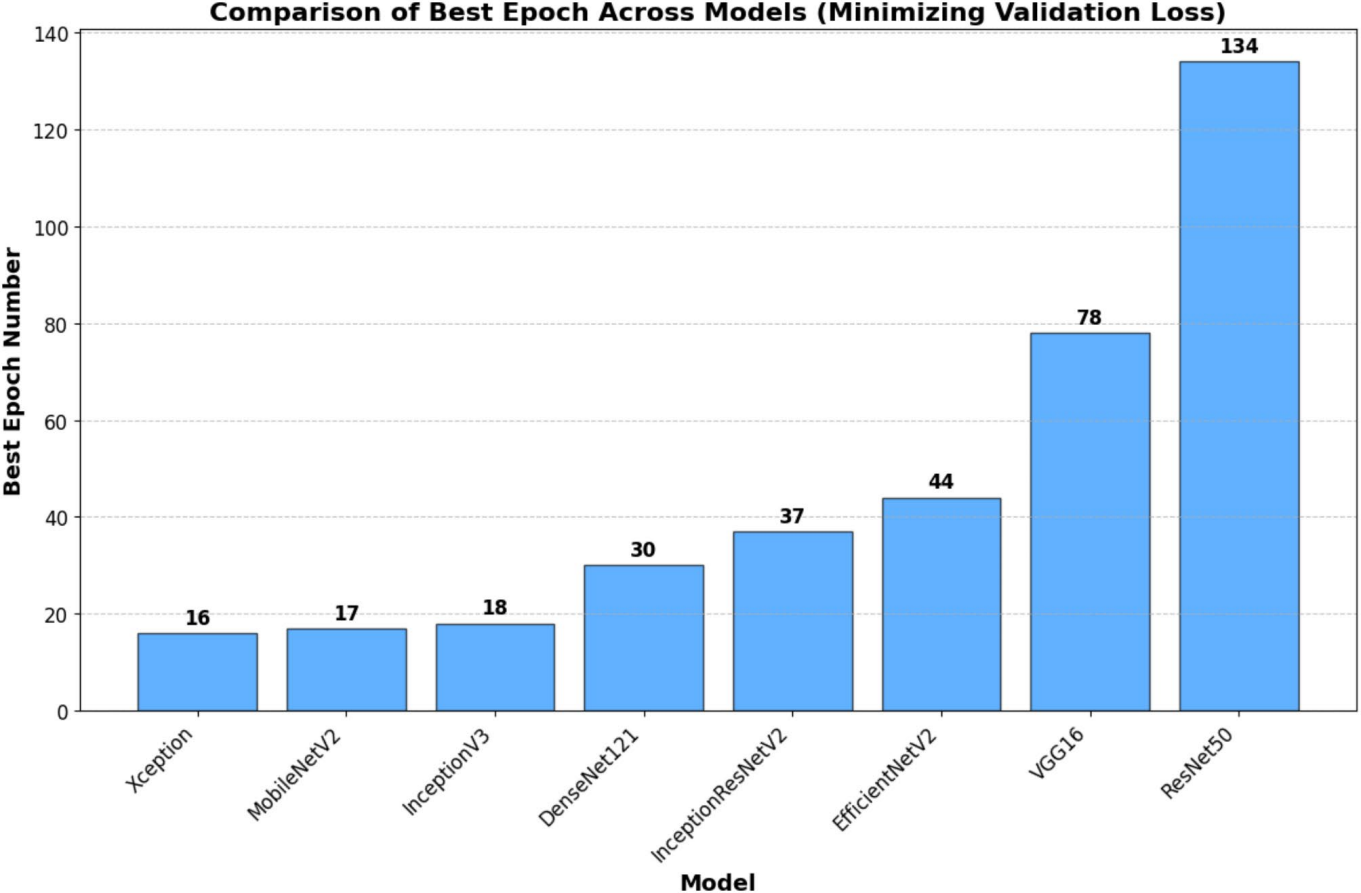
Ordered comparative analysis of optimal epoch selection in DL models for automated football foul detection.

As shown in Fig. 13, the models reached their best performance at different epoch numbers before the full 300-epoch limit. Xception reached optimal performance at epoch 16, MobileNetV2 at epoch 17, and InceptionV3 at epoch 18, demonstrating their ability to learn quickly and effectively. DenseNet121 attained peak validation performance at epoch 30, InceptionResNetV2 at epoch 37, and EfficientNetV2 at epoch 44, indicating a reasonable training length. VGG16 necessitated 78 epochs, but ResNet50 required the most time to converge at 134 epochs, signifying a greater demand for computing resources and prolonged training duration.

Since researchers want to achieve lower validation loss first and high validation accuracy second, based on this, epoch30 for DenseNet121 would be an ideal epoch to choose. This epoch yields the lowest validation loss with strong classification performance. In an analogy, one could argue that epoch 37 for InceptionResNetV2 and epoch 17 for MobileNetV2 also had a good trade-off of low validation loss and high generality. These findings highlight the significance of early stopping, allowing models to avoid overfitting and to train as optimally as possible. In terms of fast convergence and suitability for real world implementations (real time with less training data), models like Xception and MobileNetV2 can be noted as the most distinguished. In contrast, despite a successful performance, ResNet50 & VGG16 took considerably longer time to train and thus, may not be the most efficient options if computational efficiency is primary.

The visual studies show that for automatic foul identification in football DenseNet121, InceptionResNetV2, and MobileNetV2 are the most efficient DL architectures overall. These models showed better classification, generalization, and computational efficiency as they routinely performed well over several evaluation criteria.

## Discussion

### Comparative performance analysis

This study assessed eight modern DL architectures for automated foul identification in football events. Table 7 shows the relative performance over important evaluation criteria including Test Accuracy, Precision, Recall, F1-score, AUC. With a test accuracy of 87.57% and an AUC of 0.962, InceptionResNetV2 stood out among the evaluated models as the best one exhibiting great discriminating capacity between foul and non-foul events. The hybrid architecture gains from the combined strengths of the Inception and Residual Network designs, hence improving its capacity to capture local spatial features and deep hierarchical representations. Closely trailing with a test accuracy of 86.86% and the best precision (0.9786), DenseNet121 proved to be quite good at reducing false positives. DenseNet121 is especially helpful in cases where avoiding erroneous foul calls is vital since this implies that it is quite confident in its positive predictions.

With a test accuracy of 86.86% and an F1-score of 0.8889, MobileNetV2, a lightweight architecture, likewise shown remarkable performance stressing its capacity to properly balance precision and recall. This performance qualifies for use in real-time applications such VAR systems even with its reduced processing complexity. Conversely, with the lowest test accuracy (69.0%) and the lowest AUC (0.7656), EfficientNetV2 underperformed among other models. This suggests that EfficientNetV2, despite its state-of- the-art design in other fields, may suffer with the intricate and highly dynamic nature of football foul detection, maybe due to the limited spatial cues present in cropped foul detection images. Strong prospects for automatic foul detection systems, the top-performing models (InceptionResNetV2, DenseNet121, and MobileNetV2) routinely showed better balance across all measures.

### Training and validation performance

The training and validation performance, shown in Table 6, provides some important insights on the dynamics of model learning. Both DenseNet121 and MobileNetV2 achieved perfect training accuracy (1.0) and very low training loss (0.006 and 0.0086, respectively) and transpired to quickly and completely converge. Moreover, these models also had high validation accuracy (97.64% and 96.86%, respectively) and low validation loss, indicating their robustness to unseen validation data. This importance holds however for foul detection is where external factors of the environment like angles of viewing, lighting, occlusions adds significantly to the complexity.

InceptionResNetV2 in addition showed very consistent training behavior, with a training accuracy of (99.61%), low training loss (0.0191), and very good validation accuracy of (96.29%) which corresponded nicely with its strong test performance. On the other hand, EfficientNetV2 achieved a significantly lower training accuracy (66.09%) and validation accuracy (68.36%), suggesting that the model was unable to generalize well enough to learn useful representations from the dataset it was given. This performance gap may be due to its architecture, which was designed for generic image classification purposes instead of fine-grained action recognition like that of football fouls. Notably, ResNet50 took the most epochs (134) to converge, which indicates some difficulty in optimizing deep residual networks on this dataset. Nevertheless, ResNet50 reached a commendable validation accuracy of (91.71%), showcasing its ability to learn intricate visual patterns with ample training time.

### Robustness and generalization

The robustness of the models was determined according to the differences in performance between training, validation and test sets. Strong generalization was also displayed by models like DenseNet121 and InceptionResNetV2 by little performance drop between validation and test sets. This implies that these models can capture not excessively data-dependent foul-related features, improving their robustness in practical applications.

MobileNetV2, however, besides showing a quite high accuracy as well, proved to have an impressive robustness and consistent accuracy across all datasets, hinting at a potential usage in restricted resource situations or when being mobile. On the contrast, this study observes that EfficientNetV2 is not generalizing effectively here since its performance has dropped significantly from training sets to validation and test sets. Such behavior could be a sign of difficulties adjusting to the dynamic nature of football fouling or overfitting to non-representative training routines. The precision-recall balance is especially important for foul detection systems because false positives might interfere with play and false negatives could lead to missed fouls. DenseNet121 is conservative, but it’s extremely great accuracy (0.9786) and strong recall (0.8129) demonstrate that it might be employed for high-confidence foul identification. In professional settings when precision rules above coverage, this should be better. InceptionResNetV2 is a balanced choice for applications requiring both high detection rates and minimal false alarms as it earned the highest F1 score (0.8966), therefore displaying the best trade-off between accuracy and recall.

### Future work

This study’s findings highlight the promise of DL models for automatic foul detection in football, however several research directions might improve the robustness, interpretability, and practical use of these systems. A promising approach involves the integration of temporal modeling techniques, including Long Short-Term Memory (LSTM) networks, Gated Recurrent Units (GRUs), or Transformer architectures, which can capture the temporal dynamics of foul events by analyzing sequences of frames instead of isolated images. Also, multiple modalities with videos and audio commentary, references on player tracking matrices, and official comments from the referees could provide more contextual information to improve the precision of foul classification. Another important aspect to research in the future is the explainability and transparency of these models. Techniques such as GradCAM + + and other visualization tools might also provide referees and analysts with intuitive visuals explanations, thus increasing the confidence of their decision based on machine outcomes. Due to the variability in environmental conditions between stadiums, highlights, and camera set up, future work should also focus on domain adaptation and data augmentation strategies, for example simulating difficult lighting or synthetic crowd occlusion, to improve model generalization in different real-world circumstances. Moreover, to enable real-time implementation in VAR systems, it is essential to explore optimization strategies such as model quantization, pruning, and hardware acceleration to achieve low-latency inference while maintaining accuracy. Augmenting the training dataset to include footage from several leagues, distinct camera perspectives, and diverse player demographics will significantly improve the algorithms’ capacity to generalize across different match scenarios. Future endeavors should focus on the seamless incorporation of advanced foul detection systems into current VAR infrastructures, ensuring compatibility with live broadcast feeds, referee review interfaces, and instant replay mechanisms, thus facilitating efficient and transparent decision-making in professional football.

## Conclusion

This study provided the comparative performance of eight DL architectures in the attainment of automated foul detection in football, serving to provide information on the performance, robustness and potential use in a professional officiating system. It is concluded that InceptionResNetV2, DenseNet121 and MobileNetV2 represent the best trade-offs between accuracy, precision, recall and generalization capability and are good candidates for future developments and integrations into football officiating workflows. DenseNet121’s exceptional accuracy is a prime example of the unique advantages that these models offer, as it is highly beneficial for the reduction of erroneous infraction calls. InceptionResNetV2 achieves an effective balance between generalizability and detection precision. These models can be incorporated into the VAR system, which enables match officials to anticipate potential infractions and identify unclear situations. GradCAM + + heatmaps can also be used to show where the model made its decision. It would further improve decision-making accuracy and the transparency of officiating decisions, in turn raising the trust of players, coaches, and fans. To leverage these advantages, future work needs to make those models real-time deployable via techniques (for example on quantization and result optimization) as well as to enlarging training-data in order to cover the variety of leagues, camera angles and environments. Detecting such events could be further improved by incorporating temporal modeling to analyze player interactions across sequences of frames, as well as fusing visual data with complementary information like audio commentary and player tracking data. With these developments, automated foul detection systems have the potential to be a viable and essential aspect of contemporary football officiating, assisting referees in their efforts to achieve fairness and precision in decision-making.

## Data Availability

The datasets generated during and/or analyzed during the current study are available from the corresponding author on reasonable request.

## Abbreviations

AI: Artificial intelligence
AUC: Area under the curve
AUC-ROC: Area under the roc curve
CNN: Convolutional neural network
CV: Computer vision
DL: Deep learning
FPR: False positive rate
GradCAM++: Gradient-weighted class activation mapping++
ReLU: Rectified linear unit
ROC: Receiver operating characteristic
TPR: True positive rate
VAR: Video assistant referee
ViT: Vision transformer
